# The Physiology of the Circulation

**Published:** 1859-12

**Authors:** John C. Dalton

**Affiliations:** Professor of Physiology and Microscopic Anatomy


// NEW YORK MONTHLY REVIEW

AND

BUFFALO MEDICAL JOURNAL.

**VOL. 15.** DECEMBER, 1859. **NO. 7.**

### ORIGINAL COMMUNICATIONS.

ART. I.—*The Physiology of the Circulation. A Course of Lectures delivered at the College of Physicians and Surgeons, New York, in the Fall Term of* 1859. By JOHN C. DALTON, JR., M. D., Prof. of Physiology and Microscopic Anatomy.

### LECTURE II.

(SEPTEMBER 21.)

Action of the Heart.—Constant Character of Pulsations.—Form and Position of Ventricles.—Of Auricles.—Crossing of Venous and Arterial Streams in Heart.—Ventricular Valves.—How Supported.—Their Action. — Difference between Mitral and Tricuspid.—Closure of Valves from behind forward.—Question of Regurgitation.—Action of Arterial Valves.—Position of Different Parts of Heart. —Case of M Groux.—Other Similar Instances.

TO-DAY, Gentlemen, we shall pass directly to the study of the principal central organ of the circulation; that is, the Heart. This organ, though it does not constitute the only means of propelling the blood through the vascular system, is, perhaps, the most important of all. It is a central organ of impulsion for the circulating fluid, which continues its active operations, without cessation or relaxation, from the first appearance of life in the embryo, down to the last moment of adult existence. Not for one instant, during a life of sixty or seventy years, is the heart quiet; and it is certainly, therefore, the most important of the circulatory organs, in regard to the incessant and indispensable character of its functions, to be found in the whole body.

It is in the action of the heart, accordingly, that the manifestations of life continue longer than elsewhere, and that the symptoms of death show themselves most distinctly and unmistakeably. All the other parts of the circulatory system may be interfered with, and their functions temporarily suspended; but so long as the heart continues to beat, so long the remainder of the circulatory organs may be recalled to activity, and again enabled to perform their customary functions.

In some of the lower animals, for example, when under the influence of ether or chloroform, the respiratory movements may be diminished to a remarkable degree in rapidity and force, sensibility and voluntary motion entirely suspended, and even the flow of blood through the capillary vessels seriously impeded, without immediate danger to life. You may notice this partial suspension of the capillary circulation in various parts of the body, sometimes by the excessive pallor of the lips, the face, the mucous membrane of the mouth, and occasionally of the internal organs, so that the circulation in their blood-vessels seems nearly to have ceased for the time being; and yet, notwithstanding the suspension of the capillary movement in certain parts, or over the entire body, and even though respiration may have ceased, so long as the heart continues to beat it is possible that the rest of the circulatory apparatus may again be excited to activity. But let the heart stop for one moment,—let but a few of its pulsations be lost,—and, for some reason which we do not precisely understand, the animal organism can never again be reanimated, nor the operation of its functions restored.

This fact I have noticed very frequently in experiments upon the lower animals, when watching the effect of etherization upon the circulatory and respiratory organs. In the great water-lizard, for example (*Menobranchus*), while studying the phenomena of the circulation through the gills, I have seen the motion of the blood in the branchial capillaries become absolutely arrested under the influence of the ether, and continue inactive for a considerable time, the animal remaining meanwhile in a condition of profound anæsthesia; and yet recovery has taken place a few moments after the suspension of inhalation, and the animal has regained entirely its original activity, and the normal operation of its functions.

I have frequently seen, also, in dogs and cats subjected to the influence of ether or chloroform, the movements of respiration entirely cease; but so long as the beating of the heart could be felt through the ribs, however faintly, I have always anticipated with certainty the restoration of the respiratory function. Let the cardiac pulsations, however, be arrested, if only for a moment, and the fate of the animal is sealed; for no influence can restore them to action, and all the other functions depend, directly or indirectly, upon their continuance.

You will see, also, in studying the mode of its operation, that the heart is the most important of the circulatory organs, in regard to supplying the mechanical forces which assist in sustaining the movement of the blood. It is, as I have already said, a central organ of impulsion for the circulating fluid. It is a double forcing-pump, which receives the venous blood at one extremity, and sends forth arterial blood at the other. It is intermediate between the venous and arterial systems; and its function is the transmission of the blood from one to the other of these two great divisions of the circulatory apparatus.

We find that in all classes of animals which are provided with a heart, the essential points and characteristics of its anatomical structure are the same. The heart is nothing more than a special portion of the circulatory system; a hollow, muscular organ, the cavities of which are continuous with those of the remainder of the circulatory apparatus, and which differs from the blood-vessels simply in having its cavities surrounded by a considerable quantity of contractile tissue, and in being provided with valves, to regulate the flow of blood and to give it its proper direction. Whatever may be the peculiarities in external conformation of this organ, whether single, double, or multiple,—for there are some animals that have as many as sixty or seventy hearts (*Amphioxus lanceolata*),—we find that it always consists of a cavity or series of cavities, contracting by virtue of the muscular tissue which surrounds them, and furnished with valves so arranged as to open forward, and shut in a backward direction.

This double forcing-pump, then, is situated, as an organ of transmission, between the veins on the one hand and the arteries on the other. But in the human subject, and in many of the animals, the blood is not transmitted directly from the veins to the arteries by simple passage from one set of vessels to the other. There exists, between the arterial and venous systems an intermediate organ,—the lungs,—in which the blood is converted, during its passage, from venous into arterial.

The heart, therefore, in the human species and in the higher animals, is, as I have said, a double organ. In the animals of this class there are two circulations,—one, which has relation to the general tissues and organs of the body, and one which has relation to the lungs,—one circulation and one heart, by which the venous blood is transmitted to the capillaries of the lungs, and another heart and another circulation by which the blood coming from the lungs is again transmitted to the arteries of the general system.

We find, accordingly, that in these instances the heart is divided into right and left cavities or chambers;—the right chamber receiving the venous blood and transmitting it to the capillaries of the lungs, and the left chamber receiving the blood from the lungs and transmittting it to the capillaries of the general system.

This, however, is not all. For in the human subject, and in all the higher vertebrate animals, we have not only the heart divided into right and left chambers, but we have two cavities on each side, viz., the right auricle receiving the blood from the veins and delivering it into the right ventricle, and the left auricle receiving the blood from the lungs and delivering it into the left ventricle. In both cases the auricles are the receiving organs, and the ventricles the discharging organs. If we now examine the general characters of these two sets of organs, we shall find that the auricles are very different from the ventricles, both in their structure, form, and situation.

Here you have the fresh heart of an ox, from which the auricles have been removed; and you see, accordingly, the precise form of the ventricles fully exposed. You have here a mass, consisting simply of the muscular walls of the ventricles, of a conical or pyramidal form. In its natural position, the base of the pyramid looks upward and backward, while its apex looks downward and forward.

In this second specimen, which is the heart of a dog, in its natural condition, you observe the relations, in size and structure, between the auricles and ventricles. You still recognize the ventricles as presenting nearly the same appearance as in the former instance. The left ventricle is divided from the right, externally, by a narrow furrow, directed from left to right, and from above downward, in which is lodged one of the cardiac arteries. All the portion situated below and to the left of this furrow, in which the blades of the forceps are now placed, consists of the left ventricle; and all the portion situated above and to the right constitutes the anterior surface of the right ventricle. You observe, also, the thin-walled and comparatively insignificant auricles, situated above and somewhat posteriorly to the plane of the corresponding ventricles.

The older anatomists and physiologists used to regard the ventricles as constituting, by themselves, the essential portion of the heart. They were accustomed to speak of the action of the ventricles as synonymous with that of the entire organ; and they considered the auricles not so much as portions of the heart, but rather as appendages of the venous system. In this view they were undoubtedly right to a very considerable extent. You observe, in fact, that the ventricles constitute very much the larger portion of the entire mass of the heart, while the auricles are comparatively insignificant in size and undeveloped in structure. When we come to study in detail the movements of the heart, we shall find, furthermore, that the action of the auricles is very different from that of the ventricles. It is the ventricles that, by their forcible and violent contraction, actually propel the blood, and carry on the circulation. The auricles act rather as receptacles for the blood, in its passage from the venous system, and are simply intended by their contraction to fill the ventricles, while it is the latter organs that perform the principal part of the circulatory task. Such are the general characteristics and relations of the different parts composing the heart.

Now, the course of the blood, as it passes from the veins toward the arteries, through the cavities of the heart, presents one peculiarity to which I will, in the first place, call your attention. You observe, that both the right cavities (auricle and ventricle) are situated upon a plane a little anterior to that of the left cavities. The right auricle is placed above and in front, and the left auricle above and behind. The right ventricle is below and in front, the left ventricle below and behind. Both the ventricles, accordingly, are situated below, and both the auricles above. Both the auricles, as I have already remarked, serve as receptacles; the right auricle receiving the blood from the veins of the general system, the left that from the pulmonary veins. The blood, therefore, coming from the veins, enters the heart at its upper portion, by the venous orifices. It then passes, on the right side, from the auricle to the ventricle, in a downward direction; but, on arriving at the apex of the right ventricle, it immediately changes its course. It here makes a turn upon itself; and, instead of passing as before, from above downwards, begins to pass from below upward, and from right to left. It thus arrives at a portion of the right ventricle, situated at an angle with the rest and leading toward the orifice of the pulmonary artery, which has received the distinctive appellation of the "conus arteriosus." In passing from the auricle to the ventricle, accordingly, the direction of the current is from above downward; but in passing from the ventricle to the pulmonary artery, through the conus arteriosus, it is from below upward. This change in the direction of the current of venous blood takes place, you will observe, in the anterior portion of the heart.

Now, let us examine the course of the blood, as it comes from the lungs, through the left side of the heart. From the lungs it passes, by the pulmonary veins, into the left auricle. From the left auricle it passes downward, to the left ventricle. Arriving at the apex of the ventricle it again changes its course, and passes from below upward, and from left to right, toward the orifice of the aorta. It reverses, in this manner, the change of direction which takes place on the other side of the heart. We have here, accordingly, two streams passing through the organ in two opposite directions, both of them making their way, first from above downward, and then from below upward. But the venous stream turns from below upward and from right to left, in front of the heart; while the arterial stream turns from below upward and from left to right, in the posterior portion of the organ.

The two streams, therefore, venous and arterial, cross each other in the cardiac cavities, and finally emerge from the heart by its arterial orifices, in two different directions.

We will now proceed to the examination of the structure and mode of action of the *valves*, by which the various orifices of the heart are guarded. You see here several preparations, illustrating the manner in which these valves act. In the first specimen, the pulmonary artery has been injected with alcohol, from above downwards, distending its cavity and closing the arterial valves by the backward pressure of the injection. The anterior portion of the pulmonary artery has been cut away, exhibiting its three valves lying with their edges in contact, and closing completely the orifice which looks toward the ventricle. The same condition is also exhibited in the second specimen, which has been prepared in a similar manner.

In the third preparation, the right ventricle has been injected with alcohol by passing the nozzle of the syringe between the pulmonary valves, and injecting its cavity from before backward. The anterior wall of the ventricle having been afterward cut away, the tricuspid valves are shown in a closed condition, and lying across the auriculo-ventricular orifice. You look directly into the cavity of the right ventricle and conus arteriosus, and see the oblique direction which the blood must take in passing from the ventricle to the pulmonary artery. The preparation having been hardened by the action of the alcohol, all its parts retain the precise position in which they are naturally placed at the moment of the ventricular contraction.

I would invite your attention, in the next place, to the manner in which the ventricular valves are held in place, at the time of the cardiac contraction. It is plain that, when the ventricle contracts upon its contents, it must act upon the blood with a considerable compressing power; and, if there were no anatomical contrivance to prevent it, the blood, while flowing outward through the arterial orifice, would regurgitate, with just as much rapidity and force, in the opposite direction. The membranous curtains, or valves, therefore, which prevent this regurgitation, and which are so situated and constructed that they may open forward and shut backward, must also be firmly supported in their closed position. This support is differently provided for in the auriculo-ventricular valves, and in those situated at the arterial orifices. In the tricuspid and mitral valves, we find a number of slender, rounded tendons, originating from the membranous edges of the valves, which pass from above downward, and are implanted into the fleshy columns arising from the sides and apex of the heart.

In this preparation you see the manner in which the mitral valves are supported by their tendinous chords. You observe, also, that there is an inequality in the breadth of the two membranous curtains forming the mitral valve. That situated toward the left side of the auriculo-ventricular orifice is the narrower of the two, while that on the right side is broader. The broader of these two curtains is placed directly over the orifice leading to the aorta; and when the blood is passing from the auricle into the ventricle, this valve naturally falls downward and covers the entrance to the aorta, so that none of the blood passes into the aorta at this time. As soon as the ventricle contracts, however, both the valvular curtains being thrown backward to close the auriculo-ventricular orifice, the orifice of the aorta is opened, and the blood passes freely into it from the ventricular cavity.

In this bullock's heart, from which the auricles have been removed, but in which the auriculo-ventricular valves on each side have been left undisturbed, we can see the natural play of the mitral valves. The attachments of these valves are, as you see, fully exposed on their upper surfaces, owing to the removal of the left auricle; and you can look directly into the cavity of the ventricle, through the auriculo-ventricular orifice. I now place the pipe of a syringe in the aorta, with its nozzle directed toward the cavity of the left ventricle. The aortic valves have been destroyed, so that there is no difficulty in filling the ventricle with a watery injection, from the aorta backward. The pipe of the syringe being secured in this position, I now fill the ventricle with water, and you see that the mitral valves are lifted and brought together, so that the auriculo-ventricular orifice is effectually closed.

If we first fill the ventricle gradually with water, and then suddenly distend it by a forcible stroke of the piston of the syringe, you can see how completely the mitral valves prevent the escape of the fluid. The aorta is now distended by the backward pressure, and the ventricle filled to its utmost capacity; and yet the edges of the mitral valves are in perfect coaptation, and retain their natural position. The pressure is so great that the water escapes, you observe, in several thread-like streams from the cut surface, about the auriculo-ventricular opening,—the divided orifices of minute blood-vessels ramifying in the substance of the heart, and filled from the coronary arteries at the base of the aorta. By withdrawing the piston of the syringe, the mitral valves collapse, and the cavity of the ventricle is opened; and by alternately filling and emptying the ventricle in this way, the valves can be made to alternately open and shut, thus imitating their natural play in the cardiac circulation.

Although, however, in this experiment, we can show with sufficient accuracy the movements and action of the valves themselves, it does not represent exactly the means by which their closure is effected during life, in the natural course of the circulation. Here we distend the ventricles and close the valves by injecting a fluid backward from the aorta. But nothing of this kind takes place in life. In the natural condition of the circulation, the ventricle is filled from the auricle and not from the aorta. The direction of the current of the blood is constantly from behind forward, and not from before backward. It is true that if the ventricle be filled with blood while in a relaxed condition and should then contract, it would compress its fluid contents equally in all directions, and might thus be regarded as exerting a backward as well as a forward pressure. Still, this does not seem to be the mechanism by which the tricuspid and mitral valves are really closed; for the ventricle is, in point of fact, always filled in a direction exactly contrary to that which we have employed in the preceding experiment.

Some years ago Drs. Baumgartner and Hamernik, of Germany, came to the conclusion; from certain investigations directed to this point, that the ventricular valves in the natural state of the circulation, were closed by a force acting, not from before backward, but from behind forward; that is to say, that they were closed by the pressure of the blood acting in a direction from the auricle toward the ventricle, and not by its backward pressure from the ventricle toward the auricle.

This statement may at first appear somewhat singular and difficult of comprehension; but a very simple experiment will show, I think, that it is a perfectly correct one. If we take the bullock's heart, arranged as before, with the auricles removed, and the attachments of the mitral valve exposed, by injecting the ventricle gently from the aorta backward, we can, as in the former instance, partly close the mitral valves. Now, with the valves in this partly closed condition, if we were to inject water downward into the ventricle *through the auriculo-ventricular orifice*, you would expect this would have a tendency to force open the valves. In reality, however, it has a tendency to close them more completely. I now direct a stream of water from above downward upon the half-closed valves, and you see they immediately become as distinctly and perfectly closed as if the injection had been made in a backward direction from the aorta.

We can even accomplish the whole process of filling the ventricle and closing its valves, by injecting it through the auriculo-vertricular opening. If I now pour a stream of water from above into the empty ventricle, you see the mitral valves gradually float upward and approximate each other, as the cavity of the ventricle fills with fluid ; and at last a somewhat sudden and forcible injection completes the work, and leaves the valves instantly in a state of perfect coaptation.

It is in this way that the filling of the ventricle takes place in the natural course of the circulation. The contraction and relaxation of the auricle and ventricle do not alternate with each other in so regular and complete a manner as is sometimes described. The contraction of the auricle always precedes that of the ventricle, but there is still a considerable time, before the occurrence of the cardiac systole, when the ventricle and auricle are both in a relaxed condition. During this time the blood flows steadily from the large veins into the auricle, and through the wide auriculo-ventricular orifice into the ventricle. Subsequently, however, the contraction of the auricle takes place, which by a quick sharp impulse completes the filling of the ventricle, and at the same time closes the ventricular valves.

Then comes the contraction of the ventricles, which react instantly after the movement of the auricle. But you see from this, that the closure of the ventricular valves takes place not at the moment when the ventricle itself contracts, but just before that time, viz. at the contraction of the auricles; and that consequently when the ventricles react upon the blood which has passed into them from behind, their auricular orifices are already closed.

*Regurgitation.*—It has been thought by some observers that there is an essential difference in the efficiency of the various sets of cardiac valves, and in the completeness with which they guard the respective orifices at which they are situated. If we examine the mitral and tricuspid valves by the method employed above, viz. by injecting the cavity of the ventricles with water from the pulmonary artery and aorta, it is found that while the left ventricular valves (mitral) close promptly and efficiently, so as to bear a very powerful pressure without yielding, the closure of the right ventricular valves (tricuspid) is much less complete, allowing the regurgitation of a certain quantity of fluid between them.

Some years ago Dr. T. Wilson King described this peculiarity of the tricuspid valves, and attributed to it a special function for preventing overdistention of the heart, which he called the "safety-valve function of the right ventricle." Over-distention of the heart with blood would naturally have the effect of paralyzing its muscular fibres, in the same way as an excessive accumulation of urine destroys the contractile power of the bladder. Any such accumulation, accordingly, whether produced by deficient respiration, recession of the blood from the surface, or other similar cause, would be liable to produce a fatal effect by paralyzing the right ventricle, unless the exce sive distention of the organ were prevented by opening an avenue of escape for the accumulated blood. Dr. King considered this as effected by the regurgitation of the blood through the right ventricular valves. These valves are attached by means of their tendinous chords and columnæ carneæ, partly to the right side of the septum of the ventricles, and partly to the external wall of the right ventricle. This external wall is the one which most readily yields to pressure, and which, when forced outward by an accumulation of blood, or by the forcible injection of fluid, draws away with it a certain number of the tendinous chords, and so separates the edges of the valves to which they are attached.

Regurgitation will take place through the tricuspid valves, under the influence of a backward pressure, even when there is no such arrangement of tendinous chords, attached to the external or "yielding" ventricular wall, as that described by Dr. King. In the dog for instance, all the chords belonging to the curtains of the tricuspid valve are attached, either directly or through the medium of fleshy columns, to the internal or solid wall of the ventricle—that, namely, which forms the septum between the two ventricles. And yet the tricuspid valve can readily be forced in the above manner.

Here is a dog's heart with the nozzle of a syringe inserted into the right ventricle through the pulmonary artery, and secured by ligature. By driving the injected fluid into the ventricle, you observe, the ventricle is at first filled, and none of the fluid escapes. But as soon as I distend its cavity beyond a certain point, its walls are forcibly separated, the action of the ventricular valves is no longer complete, regurgitation takes place from the ventricle to the auricle, and from the auricle to the veins, and the fluid escapes from the open orifices of the venæ cavæ.

This mechanism therefore, or one similar to it, certainly exists as above described, and acts in the manner indicated when injections are employed to test the efficiency of the valves; and yet it is doubtful whether it possesses the importance which has been attributed to it in a physiological point of view, or whether the right ventricle can be regarded as exerting any peculiar and special function in providing against congestion and its consequences. It is true, that the tricuspid valves may be much more easily forced, by a backward pressure, than the mitral; but this is owing in very great measure to the fact that the left ventricle has excessively thick, muscular walls which retain their figure under pressure and maintain their valves also in position, while the walls of the right ventricle are thin, and, comparatively speaking, easily displaced by pressure. Beside, a great difference will be seen in this respect according as we examine the valves of the right ventricle when its walls are in a contracted or a relaxed condition. Immediately after death, when the walls of the ventricle are relaxed and flabby, regurgitation through the tricuspid valves may be produced with the greatest ease; since they are maintained but loosely in their position, and a little force is sufficient to separate their edges. But if the same heart be allowed to remain until cadaveric rigidity is established in its muscular parietes, the tricuspid valves will then be found to close much more readily, and to resist pressure much more efficiently than before. We can easily understand, then, that though the valves may be separated by the pressure of an injection in the dead heart, they may still be so held in place during life by the contraction of the circular muscular fibres, that no regurgitation may take place. We must recollect furthermore, that in experiments like those detailed above we operate upon the heart separated from its natural connections; which undoubtedly exert no inconsiderable influence in maintaining the proper coaptation of its parts. The normal attachments of the pericardium and the great vessels, the sustaining pressure of the lungs and of the walls of the chest, which surround the heart everywhere with a gentle and equable support, are all lost when the organ is removed from the thorax and injected separately. I have often noticed, in making these experiments, that however carefully the injection is performed, the *position in which the heart is held* has a great deal of influence on the completeness with which the valves resist pressure. In the mitral and even in the aortic valves, when fully distended by an injection, a very slight deviation of position, or undue lateral pressure on the neighboring parts, will frequently be sufficient to displace the edges of the valves, and to cause immediately a certain amount of regurgitation. The most favorable position for complete closure of the valves, which is no doubt secured during life by the normal attachments of the organ, requires to be maintained with some care in the separated heart.

It is important to observe, also, that in filling the ventricle by a backward injection from the pulmonary artery we use a mechanism for closing the valves different from that which is employed during life. In the natural condition of the circulation, as we have already seen, the ventricular valves are closed by the flux of blood passing into the ventricle from the auricle. This is true for the valves upon the right side as well as for those upon the left; and though it is true, therefore, that by an artificial injection, directed from before backward, the tricuspid valves may be forced more readily than the mitral, there seems no good reason for concluding that in the normal state of the circulation the right ventricle is not as completely protected by its valves as the left. In each case the arrangement of the valves and their support correspond with the strength of the muscular walls of the ventricles ; for in each the valves are held in place, under ordinary circumstances, by a muscular force exactly equal to that which tends to displace them by a backward pressure of the blood.

The truth is that *all* the valves about the heart are capable of displacement, and will allow regurgitation under an excessive backward pressure. This results naturally from the manner in which they are arranged. For since they are attached by their fixed edges to the circumference of the cardiac orifices they will necessarily be drawn apart and opened by too great a distention of the cavities which they are destined to close. In the left ventricle it is comparatively difficult to do this, especially in large hearts, like that of the ox, owing to the extreme resistance of the muscular parietes ; but it can, nevertheless, be accomplished by continued and forcible pressure. In the right ventricle on the other hand, such a result follows more readily, since the auriculo-ventricular opening is wider and its parietes more easily distended.

In injecting the right ventricle from before backward, the closure of the valves is never perfect until the cavity of the ventricle is tolerably well filled. Then the free edges of the valves, in a favorable position of the heart, come into apposition with each other and fold over slightly, so that they touch each other throughout a narrow portion of their borders, and the remaining parts of the valve rise in a vaulted form toward the cavity of the auricle.

If a fluid, accordingly, be forced into the ventricle in moderate quantity, it accumulates in these vaulted portions beneath the valves, and by lateral pressure forces the borders of the valves into still firmer approximation. It is only when the distention is such as actually to draw apart bodily the whole valves and their attachments, that regurgitation takes place. The injected fluid then wells up in a bubbling mass from one or two points, or, if the pressure be great, may be sent out in a fine thread-like or ribbon-shaped stream from the spot where the adjustment of the valves is most imperfect.

The aortic and pulmonary valves, gentlemen, as you are aware, are supported in a manner somewhat different from the mitral and tricuspid. In all of them, as we have seen, the valves when full have an arched or vaulted form, their convexities looking backward, and their concavities forward. But the semilunar valves of the aorta and pulmonary artery, instead of being supported by tendinous chords and fleshy columns, are held in place by the form of their free edges, which are arranged in the shape of festoons, and which, by their lateral pressure afford each other at the same time a mutual support.

Here is the aorta of an ox, separated from the heart, but with its valves and their attachments still undisturbed. By injecting the artery forcibly with water from its peripheral extremity backward, you observe its valves fill out, become applied to each other by their free edges, and form at the cardiac extremity of the vessel a thin, but very firm and resisting triple-vaulted wall, which retains completely the injected fluid, notwithstanding the distended condition of the arterial parietes.

On increasing the pressure you see that the artery becomes especially dilated at its base into three bulging prominences, or ampullæ, corresponding with the concavities of the three semilunar valves. At the same time it is to be seen, that the first effect of an increased pressure and the distention of the base of the artery is, to force the valves over farther toward each other. Their edges are thus brought into closer apposition, and greater resistance afforded to the pressure from within. You can appreciate this power of resistance by feeling the induration of the coats of the artery and of its valves at the cardiac extremity. To make the resistance of the valves and the compressing power of the arterial walls still more evident, I now hold the vessel in such a position that the valves are directed downward, and the divided extremity, into which the nozzle of the syringe was inserted, uppermost. On turning the stopcock and allowing the contained fluid a free exit, the water, you see, is thrown forcibly upward, to a height of over two feet.

It is evident, therefore, that in large animals, like the ox, where the column of blood is very heavy, and the action of the left ventricle powerful, an exceedingly severe pressure must be borne by the valves in order to maintain the natural course of the circulation. There is, accordingly, in this and some other species, a peculiarly shaped bone, situated at the base of the heart, and so placed as to support the attachments of those valves which are subjected to the greatest degree of pressure. Here is a specimen of this bone (os cardis) from the heart of the ox. It is composed, as you see, of two curved limbs, of unequal length. Its normal situation in the heart is such, that the longer of these two limbs supports on its outside the attachment of the broad curtain of the mitral valves. On its inside, one of the semilunar aortic valves is included in the space just between the junction of its two limbs, while another is partially supported by the remainder of the longer limb. The supporting function of this bone is accordingly confined almost entirely to the valves, aortic and ventricular, on the left side of the heart. Upon the right side, the force of the ventricular contraction being less, and the walls of the pulmonary artery, also, thinner and less powerful, less firmness is required in the attachments of the valves.

Let us terminate this portion of the subject, gentlemen, by some considerations relative to the position of the heart and its different parts in the interior of the chest. In most of the lower animals, the heart is nearly or quite central in position, occupying the median line. In the human subject its apex is turned to the left, so that the point of the heart strikes the walls of the chest usually at the fifth intercostal space, and a little inside a vertical line drawn through the left nipple. From constantly feeling the pulsations of the heart, therefore, in this situation, we are accustomed to speak and think of the organ as placed upon the left side of the body. Nevertheless, the heart is actually, even in the human subject, much more centrally situated than we are accustomed to believe.

The base and body of the organ are, in reality, placed in the median line, and it is only its apex which is deflected toward the left side.

It is impossible to judge of the natural situation of the thoracic organs by inspection, after the chest has been opened in the usual manner, as practiced in post-mortem examinations. For by the opening of the thoracic parietes and consequent collapse of the lungs, all the support naturally afforded by these parts being taken away, the heart necessarily falls out of position, and its normal relations are more or less deranged. This difficulty has been partly avoided, in some instances, by thrusting long needles through the integuments, in various directions, into the chest, and afterward ascertaining, on opening the thorax, what points in the various organs had been penetrated by them. This mode, however, can give only an imperfect view of the position of the internal thoracic organs, though a correct one, so far as it goes.

We may also guard against unnatural displacement of the organs, by tying the trachea in the middle of the neck before opening the chest; then the lungs are prevented from collapsing when the support of the thoracic parietes is taken away, and the heart remains in its normal situation. By an examination conducted with these precautions, we find that the ascending portion of the arch of the aorta lies nearly in the median line. The bases of the aorta and pulmonary artery, and of course the aortic and pulmonary valves, are situated almost exactly in the median line, at the level of the third costal cartilage, or the third intercostal space. The base of the aorta, where it begins to show itself above the body of the heart, is behind and a little to the right; while the upper part of the conus arteriosus and base of the pulmonary artery are placed in front and a little to the left. The aorta and pulmonary artery, accordingly, wind spirally round each other for a short distance. For, while the aorta emerges from the left ventricle behind, and thence comes forward, rising almost perpendicularly in the median line directly behind the sternum,—the conus arteriosus runs upward and toward the left, along the front of the heart, and the pulmonary artery originating from it then turns almost horizontally backward, to bifurcate and send one of its divisions (right pulmonary branch) beneath the arch of the aorta.

Of the heart proper, the most superficial, and, at the same time, most central portion, is the anterior surface of the right ventricle. This portion occupies the median line, and lies directly behind the whole width of the sternum from the level of the third costal cartilage downward to the sixth or seventh. The right auricle, on the contrary, is situated entirely to the right of the median line, sometimes even beyond the right edge of the sternum; and is placed moreover on a plane altogether posterior to that of the ventricle. The body of the auricle, in fact, does not come up at all in contact with the sternum; and it is only a portion of the appendix auricularis that overlaps, from the right, the base of the ascending aorta.

The left cavities of the heart are situated still more posteriorly, and show but little beyond the left edge of the right ventricle in an anterior view.

The following instances will show more precisely the usual position of the different parts of the heart, and the most common variations which are liable to show themselves in the human subject. In all these cases the trachea was first securely tied to prevent the escape of air and collapse of the lungs, and the sternum then removed, by two longitudinal incisions, equally distant from the median line.

Case I. In this instance, the upper lobe of the left lung, which was solidified with tubercular infiltration, covered entirely the heart and large vessels in the median line, and extended over to the right edge of the sternum.

The bases of the aorta and pulmonary artery were situated exactly in the median line, at the level of the third costal cartilage.

The ascending portion of the arch of the aorta was in the median line, from the third costal cartilage to the top of the sternum.

The appendix of the right auricle was placed quite to the right of the sternum, and behind the third costal cartilage.

The right ventricle occupied the space on each side the median line, behind the whole width of the sternum, from the third to the seventh costal cartilage.

Case II. The upper lobe of the left lung, partly tuberculous and adherent, covered the upper part of the heart and large vessels in the median line, and to the right edge of the sternum.

The bases of the aorta and pulmonary artery were in the median line at the level of the third intercostal space. The ascending aorta occupied the median line, from the third intercostal space to the top of the sternum.

The right auricle was rather more visible, in an anterior view, than in Case I., but was still placed altogether to the right of the median line,—behind the third intercostal space and fourth costal cartilage.

The right ventricle occupied the median line and whole width of sternum, from the third intercostal space to the seventh costal cartilage.

Case III. In this instance, the ascending aorta extended, in the median line, from the upper edge of third costal cartilage to the top of sternum. The conus arteriosus, with its continuation, the pulmonary artery, was situated mostly to the left of the median line, and extended obliquely upward from the third costal cartilage on the right to the level of the first rib on the left.

The appendix of the right auricle, which was full of blood, was situated entirely to the right of the median line, at the level of the second costal cartilage and second intercostal space. It overlapped the base of the aorta, but did not touch that of the pulmonary artery.

The body of the right auricle, which was very much distended with blood, projected two inches and a half to the right of the median line.

The right ventricle occupied the median space, from the third to the sixth costal cartilage. The free border of the right ventricle, at the level of the fourth costal cartilage, was one inch to the right of the median line.

Case IV. The aorta was in the median line, but lay concealed, up to the level of the first rib, between the conus arteriosus and right auricular appendix.

The appendix of the right auricle was situated in the second intercostal space, altogether to the right of the median line. The conus arteriosus was also on the second intercostal space, to the left of the median line.

The right ventricle occupied the width of the sternum, from the third to the sixth costal cartilage.

Here are a number of injected specimens, with the heart in situ and the chest laid open. In all of them you can see illustrated the principal points already mentioned. The aorta and right ventricle are situated invariably in the median line, while the right auricle is placed posteriorly and on the right side; the left border of the heart, which is formed by the left ventricle, being also turned backward, and not showing itself very prominently in an anterior view.

This question of the exact position of different portions of the heart with reference to the walls of the chest, always of interest in a practical point of view,—became very important in a case of congenital fissure of the sternum and partial exposure of the heart, which was exhibited here last year, and which attracted the attention of medical men in various parts of the country. This was the case of Mr. E. A. Groux, a man about thirty years of age, in whom there had existed from birth a complete fissure of the sternum, situated in the median line, and extending the entire length of the bone. This did not seem to be the result of a forcible division and separation of the bone, but simply of the fact that its two lateral halves, each fully formed, had never become united and consolidated with each other upon the median line. You will remember that the thoracic parietes, in the process of embryonic development, are formed simultaneously on the two sides, and grow forward until they meet in front and finally unite with each other. If their development be arrested, accordingly, toward the latter part of the process, there results a want of union between the two sides, and a permanent fissure on the median line. This fissure is more or less complete, according to the number of the different layers of tissue implicated in the deficiency.

In Mr. Groux it seemed to be only the bony tissue that was wanting. The two lateral halves of the sternum were separated from each other by a space of about one inch, at the widest part of the fissure; though by a constrained position of the limbs they might be separated to the extent of two inches. The interval between the bones was occupied by a strong fibrous layer which held the parts together, the whole being covered by perfectly healthy cutaneous investment. In the space, however, or fissure, in the median line, could be seen and felt a pulsating tumor, alternately rising and falling, and evidently consisting of some portion of the heart or its appendages.

Mr. Groux, who was a native of Hanover, in Germany, and had attracted much attention among medical men in Europe, came to this country for the purpose of exhibiting this peculiar malformation to the physicians of the United States. Though he had been examined by many hundreds of medical men, a great diversity of opinion existed among them as to what particular portion of the heart it was which produced the pulsations in the median fissure.

Some supposed it to be the aorta, very many regarded it as the right auricle, while a smaller number, among whom was Mr. Paget, of London, thought it to be the right ventricle.

A careful examination of the tumor and its pulsations, and a comparison of it with the natural position of the heart, convinced me that the last of the above three opinions was correct, and that it was the right ventricle which showed itself in the space between the two halves of the fissured bone.

The heart, it was evident, did not deviate much from its normal position in Mr. Groux, since the stroke of the apex could readily be felt at, or just above, the fifth rib, and a little inside the plane of the left nipple. The organ, therefore, was not at all displaced laterally, and was only situated a little higher than usual with respect to the ribs, a condition not unfrequent in spare and thin-chested persons.

The pulsations of the tumor, visible at the widest part of the opening, extended from the second to the fourth intercostal space, and from the median line to the left edge of the fissure. They consisted of successive undulatory movements, which corresponded in frequency with the arterial pulsations. The tumor filled up and became prominent in a direction from below upward, and from left to right. Its contraction, which was synchronous with the impulse of the heart, consisted in a rapid wavy motion, which ran from above downward, and from rignt to left.

The tumor, therefore, was situated altogether to the left of the median line. This circumstance alone would be sufficient to show that it could not be the right auricle. For the right auricle, as we have seen, in the normal position of the heart never comes up to the median line, but is situated entirely to the right of it; and in Mr. Groux's case it was also certain, as I just observed, that there was no lateral displacement of the organ, and no reason for believing the auricle to be out of its natural situation.

On the other hand, the situation of the pulsating tumor corresponded entirely with that of the right ventricle, in a heart placed a little higher in the chest than usual, and particularly with that part of it leading to the pulmonary artery and known as the "conus arteriosus." The pulsations of the tumor also, which were wavy, progressive, and peristaltic, corresponded with those of the ventricle, which have a similar character, and not with those of the auricle, which are quick, sharp, and momentary.

But one of the most positive indications of the ventricular character of the tumor was afforded by the changes produced in it by holding the breath, or by a long expiration. When Mr. Groux took a deep inspiration, followed by a long and very slow expiration, the tumor, which disappeared more or less completely at the time of inspiration, became very much enlarged toward the end of the expiration.

This enlargement took place in two directions: first, from left to right, until, at the level of the third costal cartilage, it extended quite over to the right edge of the fissure, and thence occupied the whole width of the fissure down to the fifth costal cartilage; and second, from below upward, on the left side, until it reached the level of the lower edge of the first rib.

In this condition, the tumor presented the exact shape of the upper part of the right ventricle and conus arteriosus, when distended with blood during a suspension of the breath. It is a great mistake to suppose, as we shall see in another lecture, that it is the right auricle which is exclusively, or even principally, distended by accumulation of blood during suspended respiration. The right ventricle suffers in a similar way. If the heart be exposed in a living dog, by opening the chest and keeping up artificial respiration, whenever the respiratory movements are suspended, the right ventricle and conus arteriosus enlarge and project in front of the heart, presenting a tumor precisely similar in form to that which showed itself in the median fissure in Mr. Groux, under the circumstances which I have just described.

All these appearances make it, I think, very certain that in the ordinary condition of the respiratory movements, in Mr. Groux, the pulsating tumor situated to the left of the median line and between the second and fourth intercostal spaces, was the conus arteriosus; and that during suspended respiration the upper part of the body of the right ventricle came into view, extending over to the right of the median line, and downward as far as the level of the fifth costal cartilage.

If the finger were pressed firmly into the space between the separated halves of the sternum, above the situation of the pulsating tumor, there was felt, exactly on the median line, a deep-seated, pulsating body, of a much firmer consistency than the other. This body was undoubtedly the ascending portion of the arch of the aorta. It gave to the finger the feeling of a tense, vibrating pulsation, almost entirely similar to that which is experienced by touching the aorta of a living animal, when exposed and separated by dissection from the surrounding parts.

Various other instances of fissure of the sternum have been observed, similar to that of Mr. Groux, accompanied with more or less exposure of the heart. Geoffrey St. Hilaire mentions a case in which the fissure was incomplete, existing only at the upper part of the sternum, and in which the pulsations of the aorta could be distinctly perceived in the median space. He also quotes another instance, in which the fissure was complete, extending through the whole length of the sternum, and in which the same malformation was presented by all the members of an entire family. Cruveilhier has related a remarkable case in which the fissure in the walls of the chest of a newly born infant was accompanied by complete extrusion of the heart, this organ being entirely exposed, denuded of pericardium, and yet pulsating freely for some time after birth.

We have thus, gentlemen, gone through with most of the principal considerations relative to the structure, position, and mechanism of the heart. At the next lecture we shall study the peculiarities of its action during life, and more particularly the influence exerted upon it by disturbance of the respiratory function.

